# Structure of the Glycosyltransferase EryCIII in Complex with its Activating P450 Homologue EryCII

**DOI:** 10.1016/j.jmb.2011.10.036

**Published:** 2012-01-06

**Authors:** Martin C. Moncrieffe, Maria-Jose Fernandez, Dieter Spiteller, Hiroyoshi Matsumura, Nicholas J. Gay, Ben F. Luisi, Peter F. Leadlay

**Affiliations:** 1 Department of Biochemistry, Cambridge University, Cambridge CB2 1GA, UK; 2 Max Planck Institute for Chemical Ecology, Hans-Knoell-Strasse 8, D-07745 Jena, Germany; 3 Department of Applied Chemistry, Osaka University, Osaka 565-0871, Japan

**Keywords:** GT, glycosyltransferase, LC-MS, liquid chromatography combined with mass spectrometry, Se-Met, selenomethionine, PDB, Protein Data Bank, glycosyltransferase, antibiotic synthesis, crystal structure, cytochrome P450

## Abstract

In the biosynthesis of the clinically important antibiotic erythromycin D, the glycosyltransferase (GT) EryCIII, in concert with its partner EryCII, attaches a nucleotide-activated sugar to the macrolide scaffold with high specificity. To understand the role of EryCII, we have determined the crystal structure of the EryCIII·EryCII complex at 3.1 Å resolution. The structure reveals a heterotetramer with a distinctive, elongated quaternary organization. The EryCIII subunits form an extensive self-complementary dimer interface at the center of the complex, and the EryCII subunits lie on the periphery. EryCII binds in the vicinity of the putative macrolide binding site of EryCIII but does not make direct interactions with this site. Our biophysical and enzymatic data support a model in which EryCII stabilizes EryCIII and also functions as an allosteric activator of the GT.

## Graphical Abstract

**Figure:**
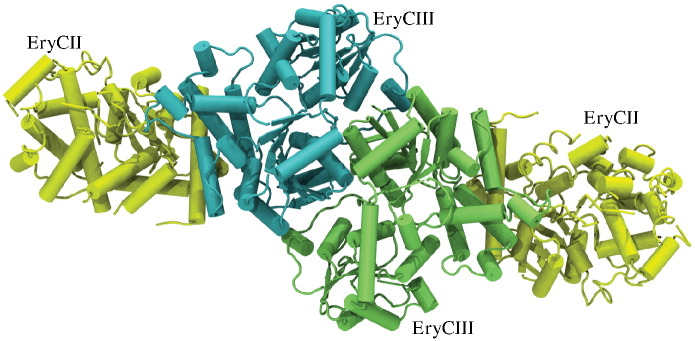

**Highlights:**

► First structure of macrolide GT and auxiliary protein. ► New system for the expression of EryCIII and EryCII. ► Auxiliary protein (EryCII) stabilizes GT (EryCIII). ► EryCII has cytochrome P450 fold but possesses an additional N-terminal helix.

## Introduction

Streptomyces and related filamentous bacteria produce a large number of structurally diverse and clinically valuable natural products, many of which require the attachment of specific deoxyhexose residues for their biological activity.^1^ Recent advances in the cloning and analysis of biosynthetic gene clusters for numerous aminoglycosides, polyketides and nonribosomal peptides have revealed the close mechanistic similarities in the pathways that lead to various activated deoxysugars and their subsequent attachment to the appropriate aglycone, catalyzed by specific glycosyltransferases (GTs). This in turn has led to the development of methods for the manipulation of such pathways to obtain novel altered natural products differing in their glycosylation status and pattern. This strategy, known as glycodiversification or glycosylation engineering,^2,3^ is crucially dependent upon the specificity of GTs and the extent to which their specificity can be altered and expanded.

A number of GTs involved in natural product biosynthesis have been structurally characterized, including GtfB, which transfers glucose from UDP-glucose to the aglycone of the glycopeptide vancomycin;^4^ the deoxyaminohexose transferases GtfA and GtfD from, respectively, the pathways to chloreremomycin and vancomycin;^5,6^ and a productive chimeric glycopeptide GT.^7^ Other structures solved include AviGT4 in avilamycin A biosynthesis,^8^ UrdGT2 in urdamycin A biosynthesis^9^ and CalG3 in calicheamicin biosynthesis.^10^ Also, the structures of two other GTs involved in antibiotic export and resistance have been reported.^11^ All of these belong to the GT-B structural superfamily of GTs, consisting of two domains, each adopting a Rossmann-like α/β fold, in which the N-terminal domain contains the acceptor binding site and the C-terminal domain encompasses the nucleotide disphospho-sugar (NDP-sugar) binding site, and catalysis occurs at the interface between them.

**Fig. 1:**
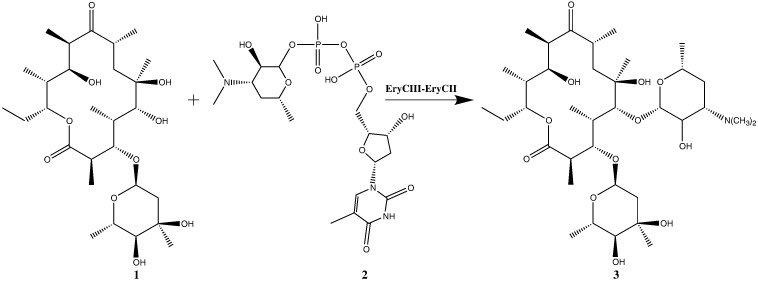
Biosynthesis of erythromycin D (**3**) from the substrates MycEB (**1**) and TDP-d-desosamine (**2**) by the GT-auxiliary protein pair EryCIII·EryCII.

Macrolide polyketides, which target protein biosynthesis and are indispensable, clinically used antibiotics, are a particularly important prospective target for glycosylation engineering, but structural information is lacking. Moreover, for a significant number of macrolide and aromatic biosynthetic GTs, the activity of the GT requires a helper protein whose function is still obscure. This dependence was demonstrated *in vitro* for the GT DesVII, which catalyzes the attachment of TDP-d-desosamine to the 14-membered macrocyclic aglycone in the biosynthesis of pikromycin in *Streptomyces venezuelae*, and whose activity was found to be dependent on the protein DesVIII.^12^ The auxiliary protein shows significant sequence similarity to cytochrome P450 but lacks the essential cysteine residue that coordinates the heme iron. Since then, at least 12 other GTs have been identified that appear to function in this way. For example, the *in vitro* activity of the GT EryCIII, which produces erythromycin D (**3**) using TDP-d-desosamine (**2**) and 3-α-mycarosylerythronolide B (MycEB) (**1**) as substrates (see Fig. 1), was enhanced by the addition of its auxiliary protein EryCII.^13^ Once exposed to EryCII, the activity of EryCIII was reportedly retained after subsequent removal of the auxiliary protein. It was suggested that the auxiliary protein permits correct folding of EryCIII into an active form but does not bind tightly and does not participate in catalysis.^13^ *In vitro* studies of the GT/helper pair AknS/AknT in aclacinomycin biosynthesis showed both partners to be readily soluble recombinant proteins when separately expressed, and AknT stimulated the glycosylation activity (using a model sugar) of AknS, but these partners likewise showed no evidence for stable complex formation.^14^ In contrast, reinvestigation of the DesVII/DesVIII pair has shown that the two proteins may associate tightly with 1:1 stoichiometry, and it has been suggested that the apparently autonomous activity of a GT after subsequent removal of the activator reflects the continued undetected presence of residual activator.^15^ It is essential to clarify the nature of the GT/helper protein interaction to aid rational engineering of these GTs. We report here the purification from recombinant *Escherichia coli* of a stable tetrameric complex of EryCIII and EryCII and the solution of the crystal structure of this complex, providing to our knowledge the first structure for any macrolide biosynthetic GT. The structural insights obtained help to rationalize the role of EryCII and of homologous auxiliary proteins in macrolide and aromatic polyketide antibiotic biosynthesis and should accelerate protein engineering efforts aimed at production of novel macrolides.

## Results

### Streptomyces chaperones increase the solubility of actinomycete GTs in *E. coli*

The expression of EryCIII in *E. coli* BL21 (DE3) codon plus RP cells yielded insoluble protein. Co-expression with the *E. coli* chaperonins GroEL and GroES yielded some soluble protein as reported previously,^16^ but purification was compromised by contaminating GroEL and GroES. In contrast, co-expression with the *Streptomyces coelicolor* chaperonins GroEL1, GroES and GroEL2^17^ produced soluble protein (Supplementary Fig. S2a) that could be purified to homogeneity. Purified EryCIII was analyzed by mass spectrometry, and a mass of 47,961 Da was obtained consistent with the loss of the N-terminal methionine residue (Supplementary Fig. S2b).

### EryCII is required for catalysis by EryCIII

**Fig. 2:**
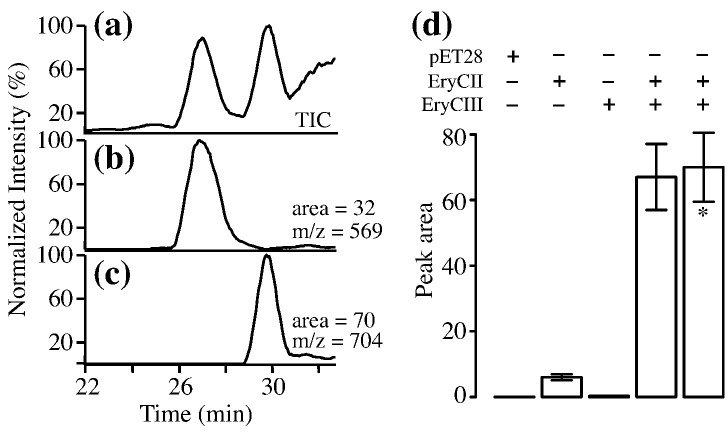
EryCII is necessary for EryCIII activity. Electrospray ionization-LC-MS analysis of the activity of co-expressed EryCIII·EryCII in cell-free extracts. Total ion count (TIC) of an ethyl acetate extract of the GT activity assay (a). Ion trace (*m*/*z* = 569) of [M + Na^+^]^+^ of MycEB (b) and (c) ion trace (*m*/*z* = 704) of [M + H]^+^ of the product erythromycin D. (d) Plot of peak areas of the erythromycin D product for the control (pET28) and extracts in which only EryCII or EryCIII were expressed are shown. The activity of a mixture of individually expressed EryCIII and EryCII is almost the same as that obtained by co-expression (∗).

Purified EryCIII was used for *in vitro* glycosylation assays as described previously,^13^ but the enzyme was inactive under all conditions tried. To confirm that added EryCII could activate the GT activity of EryCIII, we performed assays using *E. coli* cell-free extracts. Analysis of enzyme activity relied on electrospray mass spectrometric detection of the substrate MycEB and the product erythromycin D, which show characteristic *m*/*z* peaks at 569[M + Na]^+^ and 704[M + H]^+^, respectively. In Fig. 2a, a typical result from liquid chromatography combined with mass spectrometry (LC-MS) for the extract containing co-expressed EryCIII and EryCII is shown, and Fig. 2b summarizes these results. The percentage conversion for co-expressed EryCIII and EryCII, calculated from integration of the peaks on the HPLC chromatogram for both substrate and product, was 50–60%. When separate EryCIII- and EryCII-containing extracts were combined, the GT activity of EryCIII was restored, confirming as expected that EryCII is required by EryCIII. The control extract derived from cells transformed with pET28 only had no GT activity. However, the extract containing EryCII alone did have (low) enzymatic activity, suggesting that the auxiliary protein may be capable of activating endogenous GTs in *E. coli*.

### EryCII stabilizes EryCIII

**Fig. 3:**
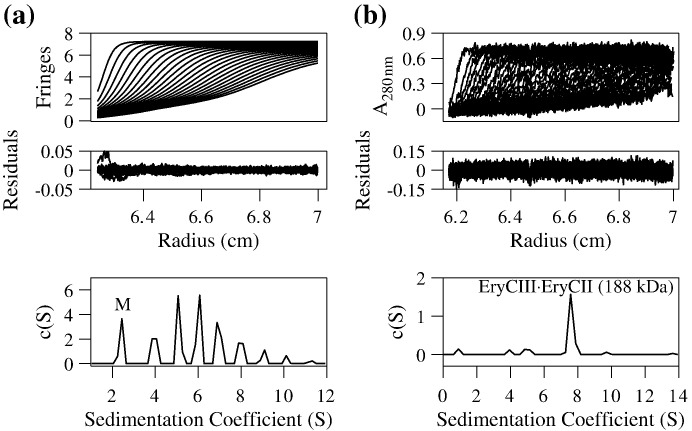
EryCII stabilizes the oligomeric state of EryCIII. Sedimentation velocity profiles, the residuals after fitting and the *c*(*S*) distribution for isolated EryCIII (a) and the complex formed between EryCII and EryCIII (b). The r.m.s.d. of the fits and the recovered frictional coefficients were 0.005, 1.7 (a) and 0.03, 1.5 (b), respectively. The sedimentation coefficient distribution of EryCIII reveals higher-order oligomers in addition to the monomeric species (*M*), which has a sedimentation coefficient of 2.44 S, while that of the EryCIII·EryCII complex consists of a single dominant component at 7.58 S corresponding to a molecular mass of 188 kDa (See also Fig. S3).

Figure 3a shows the experimental sedimentation velocity profiles of EryCIII and the co-expressed EryCIII·EryCII complex. Strikingly, the sedimentation coefficient distribution, *c*(*S*), for purified EryCIII alone has multiple peaks (2.44 S, 3.85 S, 5.07 S, 6.07 S, 6.88 S, 7.89 S, 9.10 S and 10.11 S), which correspond to monomeric EryCIII (2.44 S) and to various oligomeric forms. In contrast, the *c*(*S*) distribution for co-expressed EryCIII·EryCII (Fig. 3b) reveals only one major component with a sedimentation coefficient of 7.58 S, which, from a *c*(*M*) analysis, corresponds to a molecular mass of 188 kDa. Denaturing polyacrylamide gel analysis of purified EryCIII·EryCII (Supplementary Fig. S3) reveals bands of approximately equal intensity, suggesting a 1:1 molar ratio of EryCIII·EryCII in the complex. Taken together with the molecular masses of the individual proteins (48.1 and 40.7 kDa), the sedimentation data are consistent with a complex containing two molecules each of EryCIII and EryCII.

### Structure of the EryCIII·EryCII complex

**Fig. 4:**
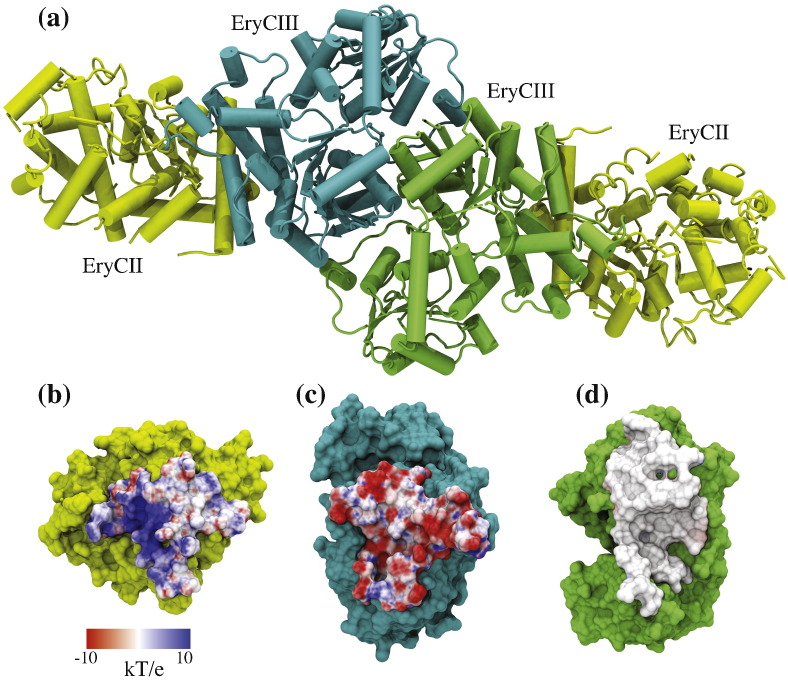
Assembly of the EryCIII·EryCII complex. The biological unit is composed of two EryCIII·EryCII dimers arranged in an almost linear array (a). The EryCIII·EryCII interface is predominantly electrostatic (b and c), while the homodimeric EryCIII·EryCIII interface is hydrophobic (d).

The structure of the EryCIII·EryCII complex was solved at 3.1 Å using data derived from crystals of both native and selenomethionine (Se-Met)-labeled proteins. Data collection, phasing and refinement statistics are summarized in Table 1. The asymmetric unit is composed of an EryCIII·EryCII heterodimer, and in agreement with the analytical ultracentrifugation data, the biological unit is tetrameric consisting of two EryCIII·EryCII heterodimers arranged in an almost linear array (Fig. 4a). Analysis of the EryCIII·EryCII tetramer by the PISA server^18^ reveals that the total buried surface area is 8568 Å^2^. The buried surface area for the EryCIII·EryCIII dimer interface is 1497 Å^2^ and that for both EryCIII·EryCII interfaces, 2787 Å^2^. The EryCIII·EryCII interface (Fig. 4b and c) is predominantly composed of electrostatic interactions between the N-terminal helix of EryCII, which occupies a groove formed by three helices [α-3/(3a), α-4a and α-6a] of EryCIII. This interface is stabilized by hydrogen bonds between the following residue pairs (EryCII·EryCIII): R26:E210, R31:A125, R31:S128, Q34:E203, Q34:P200, R37:E198, Y43:A86, R77:D89, R77:A86, R77:H85, R110:D81, L201:E203 (peptide backbone), E107:H85 and R364:H196 (peptide backbone). The EryCIII·EryCIII homodimer interface (Fig. 4d) is primarily hydrophobic but is stabilized by hydrogen bonds between A424:A46 (peptide backbone), H330:T69, V257:A70 (peptide backbone), A70:S255 (peptide backbone) and R52:H330.

### Structure of EryCIII

**Fig. 5:**
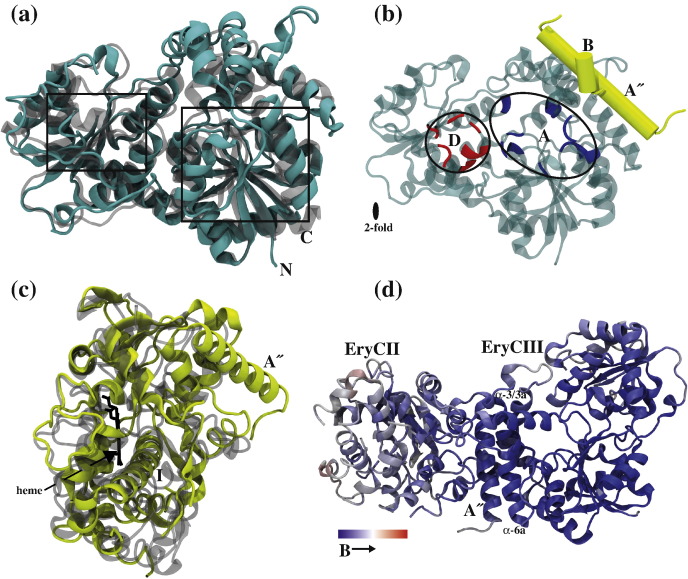
Topology and structural features of EryCIII and EryCII. (a) Superposition of EryCIII and UrdGT2 (PDB code 2P6P) reveals common features of the GT-B family of GTs. Regions containing the Rossmann-like domains are shown within rectangles. (b) Residues on EryCIII predicted to be involved in donor (d) and acceptor (a) binding are depicted in red and blue, respectively. The A″ and B helices of EryCII interact with helices that are close to the acceptor site of EryCIII. (c) Superposition of EryCII and the cytochrome P450 homologue CYP125 (PDB code 2XC3) showing the A″ helix of EryCII. Although EryCII lacks the heme moiety, the long I helix and others (J, K, K′ and L) in the vicinity of the heme-binding pocket are conserved. (d) Distribution of crystallographic *B*-factors for the EryCIII·EryCII complex. High *B*-factor values are observed for residues distal to the A″ helix of EryCII (See also Fig. S5).

The EryCIII monomer consists of two globular domains (S19–G245 and R269–G436) connected by a 23-residue (M246–R268) linker, and both are stabilized by interactions with the C-terminal helix (Fig. 5a). Through the use of the VAST^19^ and PDBeFOLD^20^ servers, close structural homologues of EryCIII were identified including CalG3,^10^ [Protein Data Bank (PDB) code 3D0R], UrdGT2^9^ (PDB code 2P6P) and the oleandomycin GTs OleI and OleD^11^ (PDB codes 2IYA and 2IYF). Of these, CalG3 is the closest homologue, and it structurally aligns with EryCIII for 318 residues with 32% sequence identity and an r.m.s.d. of 2.06 Å (Dali *Z*-score, 37; r.m.s.d., 3.0 Å). Mittler *et al.* have noted the very strong conservation of ligand binding modes in six related GTs and have successfully designed changes in the active site of the landomycin *O*-glycosyltransferase LanGT2 to create an aryl-*C*-glycosyltransferase.^9,21^ We have used a similar approach to identify active-site interactions in EryCIII. As in all these other GT-B GTs, the donor sugar binding site of EryCIII is located in the C-terminal domain. Figure 5a shows the structure of EryCIII superimposed on that of UrdGT2 (PDB code 2P6P), and Supplementary Fig. S4b shows a structure-based sequence alignment of EryCIII with several structural homologues, some with bound ligands.^10,11^ F326 of EryCIII aligns with W312 (OleI), W289 (OleD) and W285 of CalG2. In OleI and OleD, the benzyl moiety of the tryptophan side chain stacks against the uracil base.^11^ Similarly, the aromatic side chain of W285 in CalG2 stacks against the thymidine base, and an analogous interaction is expected between the nucleotide substrate TDP-desosamine and F326 of EryCIII. G275 of EryCIII is conserved in the GTs, and the equivalent residue in CalG2 (G236) interacts with the pyrophosphate oxygen atoms O2A, O3A and also C5M on thymidine. The carbonyl oxygen and amide nitrogen of V286 in CalG2 are involved in van der Waals and hydrogen bond interactions with thymidine. H342 in EryCIII is absolutely conserved in the GTs shown (Supplementary Fig. S4b), and G344 and G346 are also strongly conserved. In CalG2, the imidazole moiety of the equivalent histidine residue (H301) and the glycine residues, G303 and G305, are involved in pyrophosphate recognition. T306 of CalG2 participates in phosphate (PA, O2A) and sugar (C2′) recognition, while E309 is involved in sugar recognition only. In EryCIII, S347 and T350 correspond to T306 and E309 of CalG2, respectively. In the difference density map of the refined EryCIII·EryCII complex, residual density roughly the size of a sugar could be observed in the putative catalytic pocket, which is likely to be due to the binding of a small ligand that originates from the *E. coli* expression host. The density was not sufficiently clear to unambiguously identify the ligand; however, this observation does suggest that the pocket can accommodate a substrate.

The structure of the oleandomycin GT OleD was obtained in complex with both UDP and erythromycin A,^11^ and this similarly allowed us to identify residues in EryCIII that likely interact with the substrate 3-α-mycarosyl erythronlide B (Fig. 5b and Supplementary Fig. S4a). There are two highly conserved residues in OleD that interact with erythromycin: H19 and D329, which align with H33 and D366, respectively, in EryCIII. The imidazole nitrogen of H19 is involved in hydrogen bond interactions with O8 of desosamine. Additionally, van der Waals interactions exist between H19 and C23/C29 of the desosamine moiety of erythromycin A. Before the attachment of desosamine, H33 is therefore not expected to be involved in interactions with the scaffold. D329 of OleD is, however, involved in mycarose recognition via its interaction with C19, and this is expected to be preserved by D366 of EryCIII. The following residues in EryCIII are also expected to be involved in acceptor substrate binding by interacting with the polyketide scaffold: M127 (C21), S128 (O11), L132 (C22,C33), E151 (C35), G174 (C31), P175 (C37), D176 (C37), Q183 (C37), V222 (C34) and V223 (O13) (Fig. 5b).

### Structure of EryCII

Structural comparison^19,20^ confirms that EryCII is homologous to cytochrome P450, and the two closest structural homologues are the putative cytochrome P450 from *Mycobacterium tuberculosis*, CYP125 (PDB code 2XC3), which aligns for 264 residues with an r.m.s.d. of 2.38 Å and has 18% sequence identity, and cytochrome P450 CypX^22^ from *Bacillus subtilis* (PDB code 3NC3), which aligns for 254 residues with 20% sequence identity and an r.m.s.d. of 2.38 Å (Dali *Z*-score, 25; r.m.s.d., 2.7 Å). As expected for EryCII—and indeed the macrolide auxiliary proteins generally—the conserved cysteine is absent (Supplementary Fig. S5); thus, they are not active P450 enzymes. Structure-based alignment of EryCII and P450Cyp125 (PDB code 2XC3) is shown in Fig. 5c (see also Supplementary Fig. S5). These representations reveal that EryCII differs significantly from authentic P450 enzymes in possessing a long helix before the canonical A′ helix found in P450_*terp*_^23^ (Cyp108 from *Pseudomonas* sp.). This helix (A″) provides the main interaction surface with EryCIII in the EryCIII·EryCII complex and is expected to be present in other EryCII homologues. EryCII lacks the C and C′ helices, and parts of the G and H helices are missing. However, the I, J, K, K′ and L helices and the β-sheets in the C-terminal half are very well conserved (Supplementary Fig. S5). The portion of EryCII that corresponds to the binding site for heme cofactor and substrate in a P450 enzyme shows elevated *B*-factors (Fig. 5d), suggesting significant conformational mobility in this region.

## Discussion

The discovery that the protein DesVIII from the pikromycin biosynthetic pathway is an activator of the GT DesVII^12^ has triggered considerable interest in the mechanism of activation primarily because such glycosylation steps are crucial to antibiotic function and, if understood, should accelerate knowledge-based engineering of the attachment of novel deoxysugars to such templates. The role of proteins from the DesVIII/EryCII family could include effects on both folding and catalysis. Earlier studies of the *in vitro* properties of recombinant EryCIII encountered experimental difficulties in obtaining homogenous active enzyme^16^ but succeeded in confirming the ability of EryCII to enhance the desosaminyltransferase activity of this enzyme, as found for the DesVII/DesVIII pair. The authors of that study proposed that a stable complex between EryCIII and EryCII does not exist, based on their observation that EryCIII temporarily exposed to EryCII acquired and maintained full enzymatic activity.^13^ Reinvestigation of the *in vitro* activation of DesVII by DesVIII^15^ has shown, in contrast, that co-expression of these proteins resulted in a stable 1:1 complex, which could not be formed by simply mixing separately the purified components. Activity was also seen after temporarily exposing DesVII to DesVIII, and this was proposed to arise from residual contaminating amounts of activating DesVIII.

In the present work, we have developed a new expression system that utilizes co-expressed chaperones derived from *S. coelicolor*^17^ to facilitate the individual expression of EryCIII and EryCII and their co-expression. When co-expressed, EryCII and EryCIII form a tight complex that remains intact following nickel affinity, ion-exchange and size-exclusion chromatography. The stoichiometry of the purified EryCIII·EryCII complex in solution, determined from sedimentation velocity data, is α_2_β_2_, and this agrees exactly with the stoichiometry found in the crystal structure, where the EryCIII·EryCII complex appears as a dimer of heterodimers. On the basis of its retention volume during size-exclusion chromatography, the DesVII/DesVIII complex was previously reported to be a trimer of heterodimers (α_3_β_3_),^15^ but given the elongated shape of the complex, it is likely that this estimate was in error and other GT-auxiliary protein pairs are also α_2_β_2_ tetrameric complexes when correctly assembled.

Before the role of DesVIII was established,^12^ it was known that the function of EryCII could be replaced *in vivo* by OleP1 from the oleandomycin pathway,^24^ and since then, many examples of such crosstalk have been demonstrated.^13,15,25–28^ Thus, AknT, which has 26% sequence identity to EryCII, activates EryCIII, and EryCII, OleP1 and DnrQ, which share, respectively, 34%, 32% and 38% sequence identity with DesVIII, activate DesVII^26^ at least to some extent. However, TylMIII, which is 36% identical with DesVIII, does not activate DesVII,^15,26^ and the angolosaminyltransferase activator AngMIII, which is 45% identical with TylMIII, does not activate TylMII.^29^ Since the A″ helix on EryCII is a prominent part of the interface with EryCIII, we made an initial attempt to correlate the ability of a given activator to activate a non-cognate GT with amino acid differences in this region. We note that there are potentially some discriminating substitutions at the interface: D23 in DesVIII (R26 in EryCIII) is conserved in AknT and DnrQ (D15 and D13) but is replaced by the hydrophobic L23 in TylMIII. Similarly, L31 in DesVIII is conserved in AknT and DnrQ (L23, L21).

**Fig. 6:**
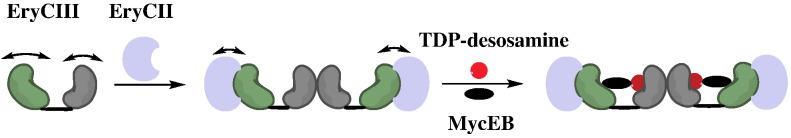
Model of a possible mechanism by which EryCII activates EryCIII. In the absence of EryCII, the dynamics of the N-terminal (green) and C-terminal (grey) domains of EryCIII are such that the acceptor (MycEB) and donor (TDP-desosamine) sites are predominantly disengaged. EryCII binding engages these sites and allows efficient glycosyltransfer. The low activity of some isolated GTs suggests that substrate binding to the enzyme may not be totally dependent on the presence of its auxiliary partner.

The hydrodynamic data for co-expressed EryCIII·EryCII strongly support the notion that one function of the auxiliary proteins is to stabilize the fold and quaternary structure of its partner GT. Additionally, the finding that the GT activity of EryCIII, expressed on its own, is almost completely rescued by the addition of soluble EryCII suggests that the auxiliary protein is able to induce a catalytically active structure in EryCIII. The EryCIII·EryCII dimer interface is formed by the N-terminal A″ helix of EryCII and helices exclusively in the N-terminal domain of EryCIII, which contains the acceptor binding site. Although this interface does not have direct interactions with the inferred acceptor binding site, it is an attractive hypothesis that allosteric binding of EryCII not only stabilizes the overall fold of the GT but also specifically modulates the structure and the dynamics of the acceptor site, thus promoting catalysis. There are several precedents for such a modulation of enzymatic activity, both by small molecules and by proteins. In a recent study of cAMP activation of protein kinase A, for example, the nucleotide was demonstrated to couple the two lobes of the enzyme by binding to a specific set of residues, the C-spine residues, which are disengaged in the absence of nucleotide.^30^ Nucleotide binding completes the spine and allows subsequent ligand binding and catalysis. Binding of EryCII to the N-terminal acceptor domain of EryCIII (Fig. 6) may likewise trigger a closer association with the C-terminal sugar-nucleotide binding domain and the correct juxtaposition of the acceptor and donor sites of EryCIII. An example of protein:protein interaction boosting enzymatic activity of one of the partners is provided by the recently solved crystal structure of a complex between the intracellular chorismate mutase of *M. tuberculosis* and the 3-deoxy-d-arabino-heptulosonate-7-phosphate synthase from the same pathway.^31^ In the complex, mutase homodimers flank a central core of synthase subunits. The synthase does not contribute directly to the mutase active site, but there are specific movements (by approximately 10 Å) of key mutase residues upon complex formation. This creates a conformation that favors catalysis, lowering *K*_m_ for chorismate by 30-fold and increasing *k*_cat_ by 4-fold. The rationale proposed for this interaction is that it allows the regulation of chorismate mutase by feedback mechanisms. It is tempting to speculate that the complex of EryCIII with its activator protein might form part of an analogous regulatory system.

Our work reveals an unusual quaternary structure for the complex of a GT and its auxiliary partner. We suggest that the heterotypic interactions of enzyme and partner allosterically activate the catalytic activity. In the case of EryCII, the fold is an elaboration of a P450-like structure and is likely to have originated from a peroxidase-like ancestor. Having lost the heme group in the central core, the protein may be more conformationally dynamic, as suggested by the distribution of crystallographic thermal disorder parameters. However, it serves as a scaffold that presents a new α-helix that forms an extensive contact surface with the EryCIII partner and which likely affects the organization of that subunit's active site. Other GTs that share the requirement for auxiliary proteins are likely to have a similar mode of activating interaction.

## Materials and Methods

The generation of plasmids for the expression of EryCIII and EryCII and the methods used to purify the resulting proteins are detailed in Supplementary Experimental Procedures. Cell pellets from  500-ml cultures were resuspended in 5 ml of a solution containing 50 mM sodium phosphate, 5% v/v glycerol and 1 mM DTT, pH 8.0, and sonicated (Mixonix-ultrasonic XL2020). The cellular debris was removed by centrifugation, and the total protein concentration was measured using the Bradford assay^32^ with bovine serum albumin as standard. Samples were concentrated to 5–6.5 mg/ml and used for activity assays without further purification. Purified proteins for activity assays were prepared as above with the omission of 2 mM DTT in the lysis buffer. The samples were loaded by gravity flow onto a  20-ml Econo-Pac chromatography column (Bio-Rad) containing 3 ml of Ni-NTA resin (Qiagen) previously equilibrated with 10 ml of a solution containing 50 mM sodium phosphate, 300 ml NaCl and 5% v/v glycerol, pH 8.0 (Buffer A) The resin was washed with 8 ml Buffer A followed by two washes with 8 ml Buffer A containing 20 and 35 mM imidazole, respectively. Protein was eluted with 5 ml Buffer A containing 150 mM imidazole. The eluted proteins were analyzed by SDS-PAGE and DTT was added to a concentration of 1 mM and the samples were used directly for activity assays.

### Activity assays

Activity assays using cell-free extracts were performed in 50 mM phosphate buffer, pH 8.0, containing 1 mM DTT and 5% glycerol, as these are the optimal conditions.^16^ The reaction mixture contained 20 μg TDP-d-desosamine (**2**), 10 μg MycEB (**1**) and a protein concentration of 6 mg/ml in a total volume of 6 ml. Protein concentrations were 0.05 mg/ml (EryCII and DesVIII), 0.56 mg/ml (EryCIII) and 0.14 mg/ml for co-purified EryCII·EryCIII. When combining EryCIII with EryCII or DesVIII, the final reaction volume was 10 ml. Reactions were incubated at 25 °C for 8–12 h,  and 100 μl of 5 N NaOH was added if required. The reactions were extracted with 1 volume of ethyl acetate, and the organic phase was recovered. Solvent was evaporated using a nitrogen stream, and the residue was resuspended in 200 μl methanol. The identity of erythromycin D (**3**) (703.45 g/mol) was confirmed using LC-MS on a Finnigan MAT LCQ equipped with an electrospray ionization-MS detector, by comparison with an authentic reference standard. Chromatographic separation was performed using a Phenomenex Synergy polar RP microbore column at a flow rate of 0.3 ml/min, with solvents A, H_2_O and 0.1% trifluoroacetic acid, and B, MeCN and 0.1% trifluoroacetic acid. The HPLC program was as follows: 100% solvent A for 3 min followed by a linear gradient from 0% to 100% solvent B in 27 min and, finally, 100% solvent B for 5 min. Data processing was performed using Xcalibur (Thermo Scientific).

### Analytical ultracentrifugation

Sedimentation velocity experiments were performed using a Beckman Optima XL-A analytical ultracentrifuge equipped with absorbance and interference optics. A solution (400 μl) of EryCIII (4.4 mg/ml) in 10 mM sodium phosphate, 2 mM DTT and a complex of EryCIII·EryCII in 2 mM DTT were placed in the sample compartment of an epon double-sector centerpiece, and 405 μl of the same solution without protein was placed in the reference compartment. The samples were centrifuged at 293 K and 50,000 r.p.m. (201,600***g***) using an An60-Ti rotor. Scans were acquired at time intervals of 1.5 min. The calculated partial specific volumes of 0.734 and 0.728 mg/ml for EryCIII and EryCII were obtained from their respective amino acid compositions using SEDNTERP,^33^ and these were used to calculate the weight-average partial specific volume of the EryCIII·EryCII complex. *c*(*S*) and *c*(*M*) distributions were calculated using the program SEDFIT.^34^

### Crystallization and structure determination

Crystals of the EryCIII·EryCII complex were obtained using the hanging-drop vapor diffusion method at room temperature and a protein concentration of 3–5 mg/ml. The well solution contained 4 M sodium formate and was mixed in a 1:1 ratio with protein solution. Crystals had cubic morphology and were flash frozen in liquid nitrogen. Native data sets were collected at ID-14.1 (European Synchrotron Radiation Facility, Grenoble), and Se-Met data sets were collected at the Diamond Light Source, (Didcot, UK). Data reduction and scaling were performed using XDS^35^ and Scala.^36^ Initial phases from the Se-Met data were obtained using autoSHARP,^37^ and a partial (30%) model of EryCIII was obtained using Buccaneer.^38^ Further rounds of manual model building and refinement were performed using Coot^39^ and Buster,^40^ and final refinement was done using PHENIX.^41^

### Accession numbers

The refined coordinates and structure factors have been deposited in the PDB with accession number 2YJN.

## Figures and Tables

**Table 1:** Crystallographic data collection and refinement statistics

	Native	Se-Met (peak)
*Data collection*		
Space group	*P*23	*P*23
Cell dimensions		
* a* = *b* = *c* (Å)	141.9	143.0
Resolution (Å)	19.7–3.1	20.0–3.5
*R*_pim_ (%)	3.6 (27.5)^a^	3.2 (14.0)
*I*/σ*I*	14.5 (3.0)	21.7 (5.8)
Completeness (%)	99.4 (100)	99.6 (100)
Redundancy	7.2 (7.5)	11.8 (12)

*Refinement*		
Resolution (Å)	3.1	
No. of reflections	17,457	
*R*_work_/*R*_free_	0.21/0.26	
No. of atoms		
Protein	5444	
Average *B*-factor	93.7	
r.m.s. deviations		
Bond lengths (Å)	0.014	
Bond angles (°)	1.862	

*Validation*		
Ramachandran favored/allowed (%)	94/100	
Poor rotamers (%)	0.2	
MolProbity score	2.1^b^	

a Numbers in parentheses are for the highest-resolution shell.

b 99th percentile.
