# Supplementary material for: Structure of the Glycosyltransferase EryCIII in Complex with its Activating P450 Homologue EryCII
Source: J Mol Biol. 2012 Jan 6;415-20(1):92–101. doi: 10.1016/j.jmb.2011.10.036 (PMC3391682; doi:10.1016/j.jmb.2011.10.036)
Supplement: Supplementary file 1 — Supplementary materials [file mmc1.pdf]

Supplementary Data

Plasmid generation

Genes encoding the GroEL1, GroEL2 and GroES chaperones were amplified using genomic DNA from *Streptomyces coelicolor* A3. Genes encoding EryCII and DesVIII were amplified from genomic DNA of *Saccharopolyspora erythraea* NRRL2338 and *Streptomyces venezuelae* ATCC15439 respectively. The *Streptomyces coelicolor* chaperones were amplified using the following primer pairs (5′ → 3′); GroEL1: GGAATTC**CATATG**GCGAAGATCCTGAAGTTCGACG and GGC**GGATCCACTAGT**TCAGTGGGAGTGGCCGTGGCTGT; GroEL2:GGAATTC**CATATG**GCCAAGATCATCGCGTTCGACGAGGAGG and CG**GGATCCACTAGT**GATCAGAAGTCCATGTCACCACCCGGC; GroES:GGAATTC**CATATG**ACGACCACCAGCTCCAAGG and CG**GGATCCACTAGT**TTTCACTTCTCGACGATCGC. EryCII was amplified using CCG**CATATG**ACCACGACCGATCGCGCCGGGCT and CCG**AAGCTTACTAGT**TCAGAGCTCGACGGGGCAGCGGTTGGT. *pfu* DNA polymerase was used in all PCR reactions and the first primer in each pair was used to introduce the NdeI restriction site. The second primer pair for GroEL1, GroEL2 and GroES introduced SpeI and BamHI restriction sites (bold) into the PCR products while for EryCII and DesVIII, SpeI and HindIII restriction sites (bold) were introduced. GroEL1, GroEL2 and GroES were cloned into pET-29a(+) as NdeI-BamHI fragments to yield L1-pET29, L2-pET29 and S-pET29 plasmids. EryCII and DesVIII were cloned into pET-28b(+) as NdeI-HindIII to yield CII-pET28 and DesVIII/pET28 respectively. EryCIII was isolated from plasmid pSGCIII[1] and cloned into pET-28b(+) as an NdeI-HindIII fragment to yield CIII/pET28. All constructs were sequenced to ensure that errors were not introduced during PCR. The L1SL2/pETcoco-2 plasmid for the co-expression of *S. coelicolor* chaperones was constructed as follows. GroEL1, GroEL2 and GroES genes were clustered by cloning them sequentially as XbaI-SpeI cassettes into pET-29a(+). In the resulting plasmid, L1SL2/pET29, each gene is preceded by a Shine-Dalgarno sequence and a ribosome binding site. The cassettes were then moved together in a single cloning step to the NheI restriction site of the pETcoco-2 expression vector to generate plasmid L1SL2/pETcoco-2 (see Figure S1a). Plasmid CIICIII/pET28 for the co-expression of EryCII and EryCIII was constructed by removing the EryCII gene from plasmid CII/pET28 with XbaI-SpeI and then cloning into the XbaI site of plasmid CIII/pET28 (Figure S1).

Protein expression and purification

E. coli BL21 (DE3) codon plus RP strain containing the plasmid L1SL2/pETcoco-2 were independently transformed with pET-28b(+), CII/pET28, CIII/pET28, CII-CIII/pET28. Cells were grown at 310 K in 2×TY media supplemented with 50 μg/ml ampicillin, 30 μg/ml kanamycin, 34 μg/ml chloramphenicol and 0.2% w/v glucose) to an OD_{600} of 0.6–0.8. Cells were then cooled on ice and induced with 0.5 mM IPTG and grown at 303 K for an additional 6 h or alternatively, at 293 K overnight. Cells were harvested by centrifugation and frozen in liquid nitrogen. For the production of Se-Met labelled EryCIII·EryCII, cells were grown in M9 minimal media supplemented with Se-methionine as described previously[2].

The cell pellet from 2–3 l of culture was resuspended in 30 ml 50 mM sodium phosphate, 300 ml NaCl 5% v/v glycerol pH 8.0 (Buffer A) and lysed using an Emulsiflex. After lysis 0.1% v/v Triton X-100 was added and the lysate clarified by centrifugation at 15,000 r.p.m. The clear lysate was loaded onto a 5 ml Hi-Trap Ni-NTA column (Amersham Biosciences) previously equilibrated with same solution. The column was washed with 10 column volumes of Buffer A including 20 mM imidazole and protein eluted with a linear gradient (20–500 mM) imidazole. Fractions were analysed by SDS-PAGE, pooled, and dialysed against 5 l of 50 mM sodium phosphate buffer pH 8.0, 2 mM DTT, and 10% v/v glycerol. Protein was concentrated and further purified by MonoQ (GE Healthcare) ion exchange chromatography followed by size exclusion chromatography using a Superdex 200 HiLoad (16/60) column (GE Healthcare) equilibrated with 10 mM sodium phosphate buffer pH 8.0, 150 mM NaCl, 1–2 mM DTT, at a flow rate of 0.5 ml/min. The eluted protein fractions were analysed by SDS-PAGE, pooled and concentrated to 4–8 mg/ml.

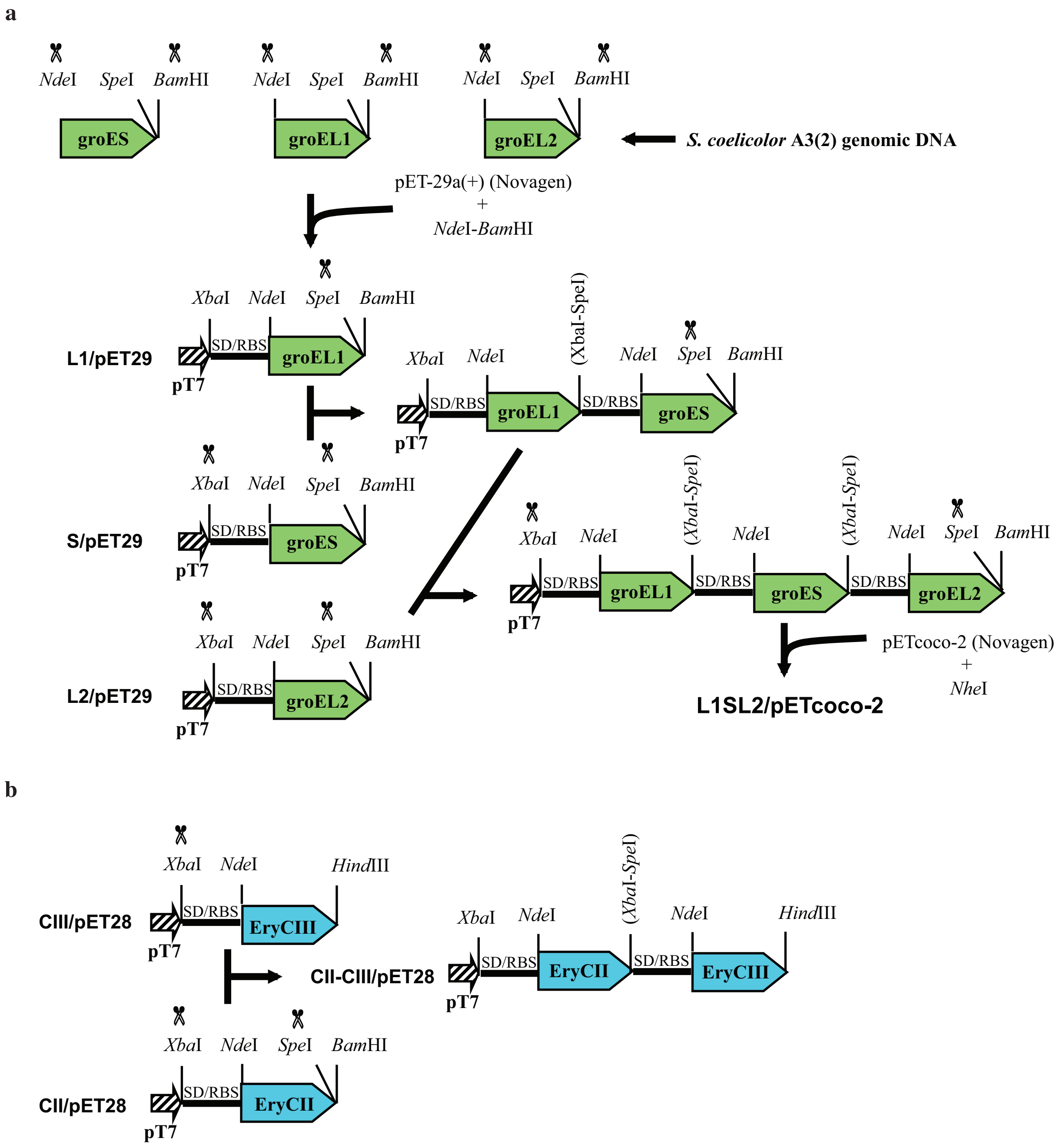

Figure S1 Construction of plasmids for the expression of EryCIII and EryCII. (a) The L1SL2/pETcoco-2 plasmid containing chaperones derived from *Streptomyces coelicolor* and (b) plasmids for the expression of EryCIII and EryCII and their co-expression.

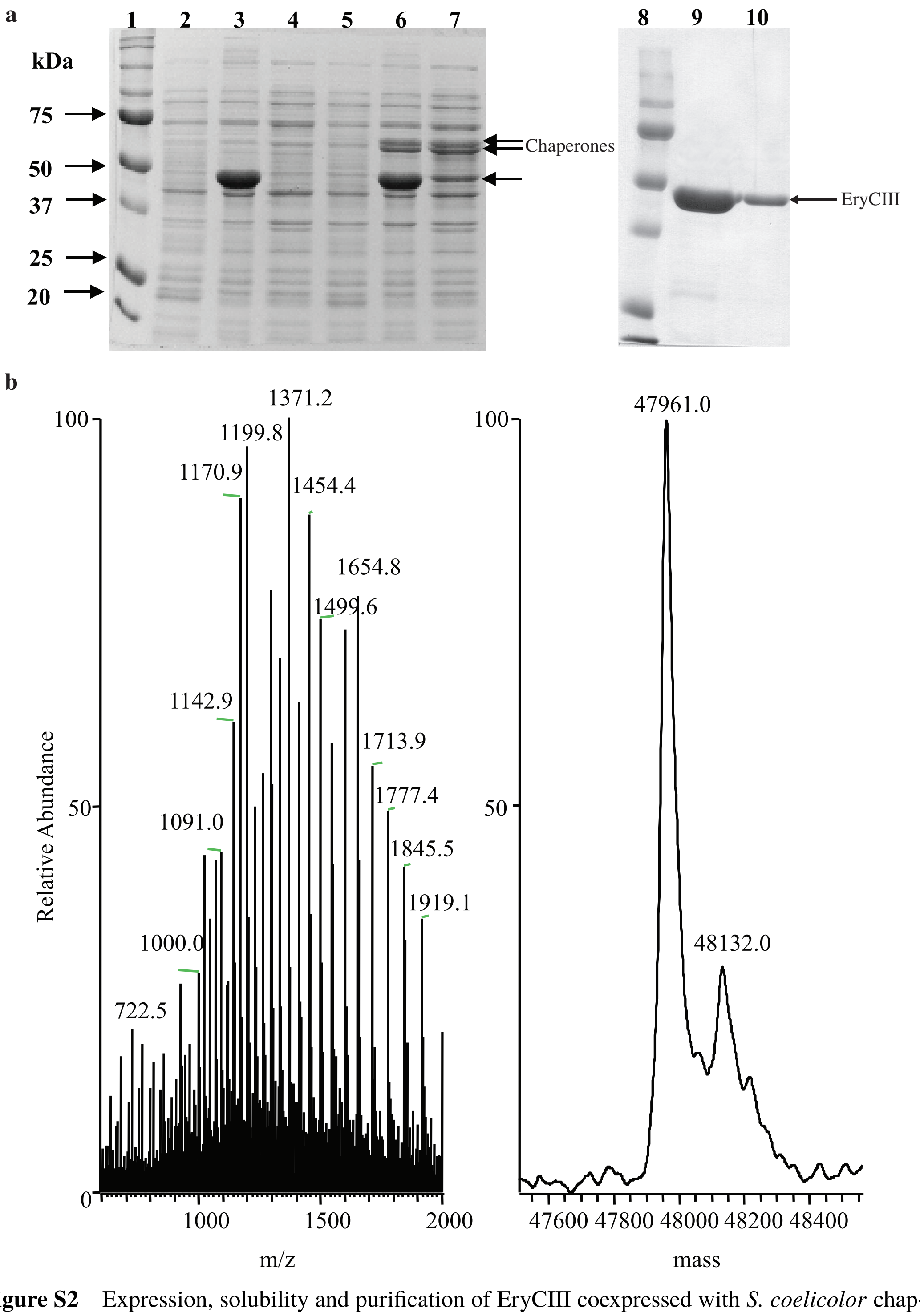

Figure S2 Expression, solubility and purification of EryCIII coexpressed with *S. coelicolor* chaperones cloned into pETcoco-2. (a) Lanes **2-4** pETcoco-2 only: pre-induction, total protein, soluble protein. Lanes **5–7, 9–10** L1SL2/pETcoco-2: pre-induction, total protein, soluble expressed protein, EryCIII eluted at 150 mM and 250 mM. (b) ESI-MS spectrum and the deconvoluted mass of EryCIII.

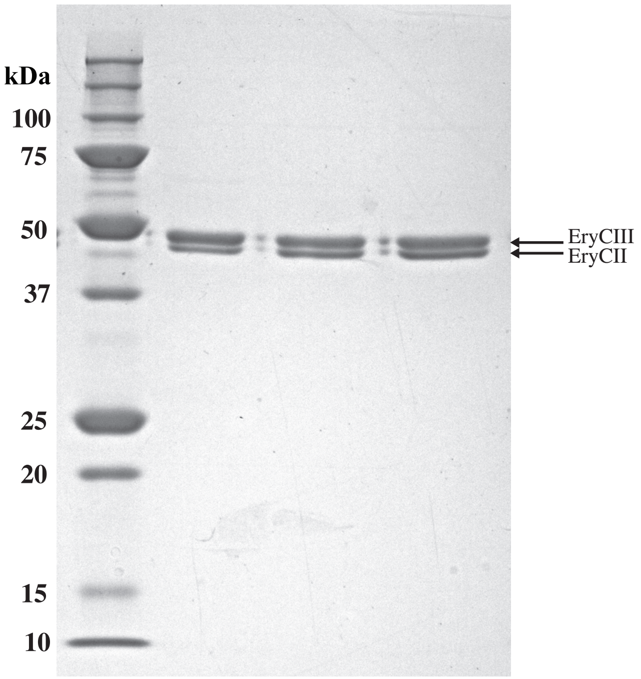

Figure S3 SDS-PAGE of the purified EryCIII·EryCII complex obtained by co-expressing the proteins as described. The lanes are the fractions obtained after size affinity chromatography.

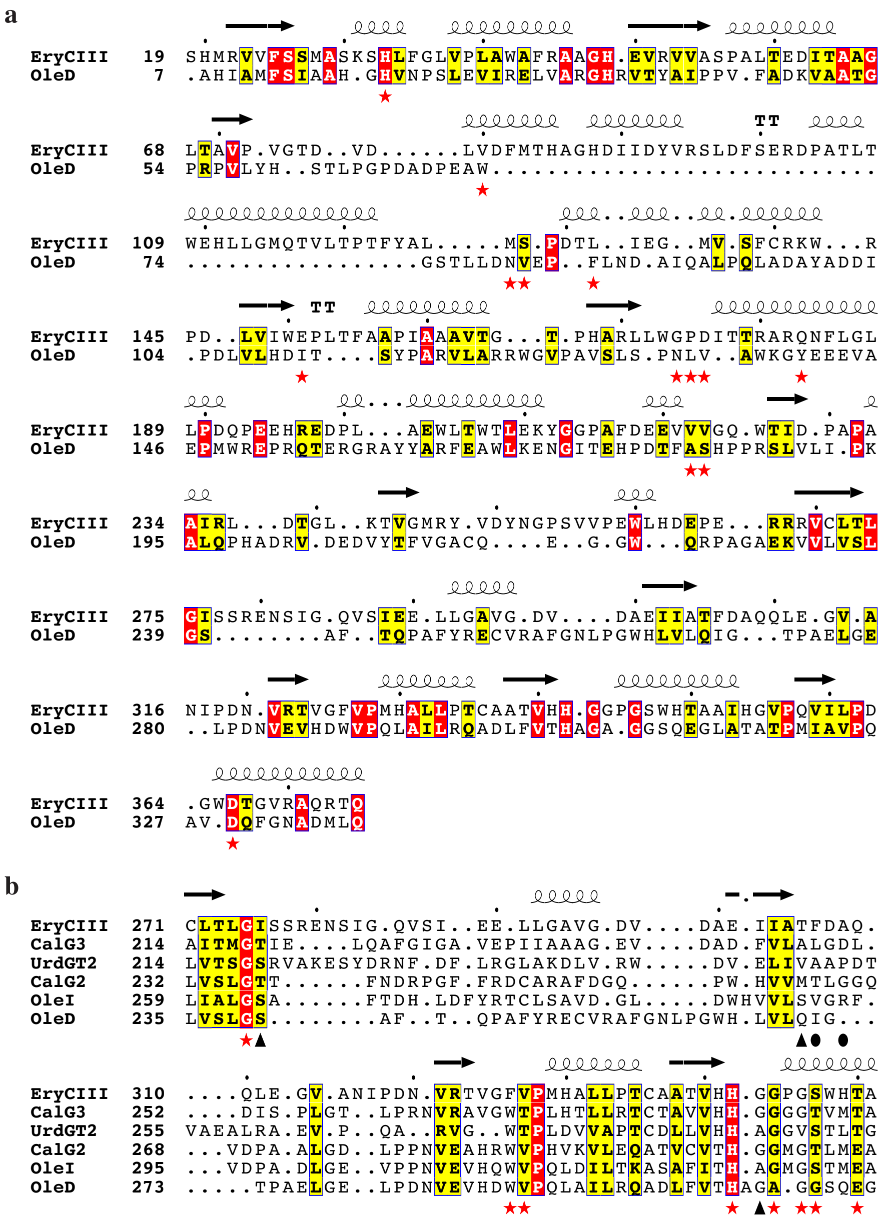

Figure S4 Secondary structure alignment of EryCIII highlighting residues involved in acceptor (a) and donor nucleotide binding (b). Red stars in panel (a) are the residues involved in erythromycin binding and in panel (b), the residues involved in both TDP and UDP binding. Black triangles and circles denote residues involved in only UDP or TDP binding.

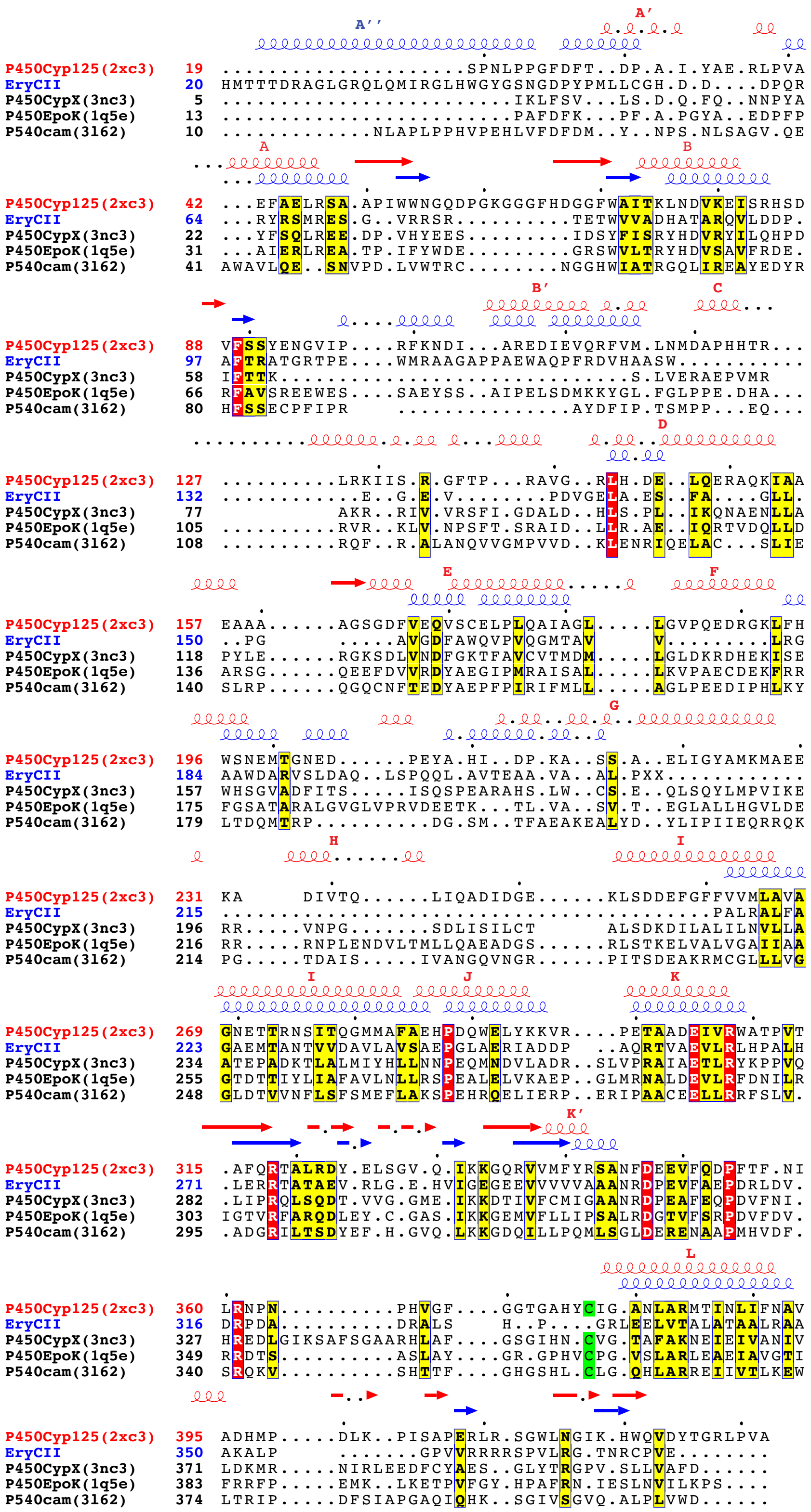

Figure S5 Secondary structure alignment of EryCII with close cytochrome P450 homologs. Note the presence of the A′′ helix in EryCII which is absent in the other family members. The helices are labelled following the convention of Hasemann *et al.*[3] Conserved cysteines are shown in green.

References

1 Gaisser, S., Reather, J., Wirtz, G., Kellenberger, L., Staunton, J. & Leadlay, P. F. A defined system for hybrid macrolide biosynthesis in *Saccharopolyspora erythraea*. *Mol Microbiol* **36**, 391–401 (2000).

2 Duyne, G. D. V., Standaert, R. F., Karplus, P. A., Schreiber, S. L. & Clardy, J. Atomic structures of the human immunophilin FKBP-12 complexes with FK506 and rapamycin. *J Mol Biol* **229**, 105–24 (1993).

3 Hasemann, C. A., Kurumbail, R. G., Boddupalli, S. S., Peterson, J. A. & Deisenhofer, J. Structure and function of cytochromes P450: A comparative analysis of three crystal structures. *Structure* **3**, 41–62 (1995).
